# The Impact of Early Postnatal and Juvenile Social Environments on the Effects of Chronic Intranasal Oxytocin in the Prairie Vole

**DOI:** 10.3389/fnbeh.2019.00206

**Published:** 2019-09-13

**Authors:** George S. Prounis, Alexander G. Ophir

**Affiliations:** Department of Psychology, Cornell University, Ithaca, NY, United States

**Keywords:** intranasal oxytocin, early-life social experience, alloparental care, partner preference, prairie voles, *Microtus ochrogaster*

## Abstract

Interactions between social experiences at different stages of development (e.g., with parents as juveniles and peers as subadults) can profoundly shape the expression of social behavior. Rarely are the influences of more than one stage of developmental sensitivity to social environment investigated simultaneously. Furthermore, oxytocin (OT) has an extraordinary effect on a breadth of social behaviors, activationally or organizationally. The use of intranasal OT (IN-OT) has become increasingly common therapeutically in humans and scientifically in non-human experiments, however very little attention has been paid to the potential developmental consequences on social behavior that might result. We investigated the effects of early-life social environments and the impact of chronic IN-OT on social behavior at different stages of development in male prairie voles (*Microtus ochrogaster*). We raised animals under two conditions: “socially enriched” (in which they were biparentally reared and then weaned into group housing as subadults), or “socially limited” (in which they were reared by a single-mother, and that were then weaned into social isolation). Males raised under each condition were either administered daily doses of IN-OT or a saline control for 21 days from postnatal day (PND) 21–42. During this time, we assessed the prosocial behavior subjects demonstrated by evaluating juvenile affiliation (as subadults), alloparental care (as adults no longer being exposed to IN-OT), and partner preference tests to assess tendencies to form adult monogamous pairbonds. We found that “socially limited” males, exhibited increased social contact in juvenile affiliation tests at PND 35 and 42. These males were also more likely to form a partner preference than “socially enriched” males and formed stronger partner preferences overall. IN-OT did not alter these behavioral effects. We also found that “socially limited” males exhibited a distinct response to chronic IN-OT treatment. When compared to all other treatment groups, “socially limited” males that received IN-OT exhibited a greater amount of huddling behavior in the alloparental care test. This effect was, in part, explained by an absence of attack behavior, found only in these males. This study contributes to understanding the complex interactions between the developmental social environment, oxytocin, and social behavior.

## Introduction

Social environments can vary tremendously across stages of development and profoundly shape the social behavior of an individual (Tzanoulinou and Sandi, 2017). Oxytocin (OT) is often implicated as a major regulator of social behavior in human and non-human species, and as a mechanism that shapes social development (Neumann, 2008; Rilling and Young, 2014; Feldman, 2015a,b; Walum and Young, 2018). Therefore, one way in which early-life social experience can have long-term consequences on social behavior is through developmental effects on the OT system. However, it remains unclear how social experiences at different stages of development impact adult behavior, and if and how social experience at different developmental stages might interact. Furthermore, the therapeutic use of OT for some children is becoming increasingly common and it remains unclear how the exogenous delivery of OT might further alter the complex nature of social development. In the current study, we briefly discuss each of these points and ask to what extent juvenile (i.e., subadult) and adult social behavior are altered by: (i) social environments experienced during two stages of development; (ii) by the non-invasive treatment of OT; and (iii) the potential interactions therein.

Specific features of social environments during critical developmental periods of life, such as interactions with parents or peers, could induce natural changes to the oxytocin system by altering OT synthesis or release, and/or OT receptor (OTR) density. Indeed, rats that receive higher amounts of maternal care develop increased densities of OTR in important parts of the brain that regulate social behavior, and they exhibit higher maternal care as adults (Francis et al., 2000, 2002; Champagne et al., 2001). On the other hand, rats experiencing routine maternal separation express similar brain region-specific increases or decreases of OTR and exhibit increases in anxiety and aggression (Kalinichev et al., 2002; Veenema, 2009; Lukas et al., 2010). In bi parental species, such as the mandarin vole and prairie vole, removal of fathers from the family unit alters OTR development and impairs social cognition (Cao et al., 2014; Prounis et al., 2015), and this can also reduce alloparental care and retard establishment of partner preferences (Ahern and Young, 2009). Interestingly, evidence has indicated that male and female prairie voles equally contribute to offspring care (Thomas and Birney, 1979) and single mothers do not appear to modify the amount of licking and grooming directed towards pups if fathers are removed (Ahern and Young, 2009). Together, this suggests that some of the OT-mediated behavioral effects on developing offspring just discussed could be attributable to the total reduction of care they received.

Social factors beyond the natal environment can also shape the OT system and behavior during juvenile and adolescent development. For example, male prairie voles that live in a socially and spatially enriched environment after weaning develop higher densities of OTR in many regions of the forebrain (Prounis et al., 2018). Similarly, mice that were exposed to high levels of early postnatal peer interactions later showed enhanced adult affiliative behavior and greater OTR density within the amygdala (Branchi et al., 2013). On the other hand, social isolation as subadults (the life-stage between weaning and adulthood) alters OTR receptor density (Prounis et al., 2015), and promotes anxiety (Pan et al., 2009) and depressive-like behaviors (Grippo et al., 2007b) in prairie voles.

Importantly, the impact of social environments during perinatal and subadult stages of development can interact. The quality of maternal care behavior received during perinatal development and environmental enrichment during subadult development interactively shape OTR expression and maternal behavior in rats (Champagne and Meaney, 2007). Furthermore, male prairie voles that are reared by a single-mother and then later experience isolated housing, demonstrate increases in lateral septum (LS) OTR and an impairment to social recognition (Prounis et al., 2015). Taken together, the aforementioned examples demonstrate that oxytocin is not only a key regulator of social behavior, but it is highly sensitive to socio-environmental influences, laying the groundwork to develop a deeper understanding of how variable social environments impact the development of social behavior.

Chronic exposure to chemical factors over development can also impact social behavior in a developing animal (e.g., Trezza et al., 2014; Davis et al., 2019). For instance, it is well-established that administration of exogenous chemicals (even those with endogenous sources) can compensatorily reduce ligand and/or receptor expression, in turn impacting neural function and behavior. OTRs are no different; persistently agonist-stimulated OTRs will desensitize, internalize and downregulate (Gimpl and Fahrenholz, 2001). Such phenomena raise important questions about how chronic early-life administration of OT could affect the neural development of animals, especially considering the common practices of administering therapeutic drugs, including OT, to children. Not surprisingly, exogenous manipulations of OT during early development affect social behavior later in life (Bales and Perkeybile, 2012). For example, intraperitoneal (i.p.) exposure to OT on postnatal day (PND) 1 facilitates adult male partner preference formation, whereas exposure to an OT antagonist reduces alloparenting in males (Bales and Carter, 2003; Bales et al., 2004). Moreover, i.p. injections of OT in neonatal prairie voles lead to dose-specific changes in alloparental care and attachment behaviors (Bales et al., 2007b). Oxytocin i.p. injections reverse the effect of social isolation on depressive-like behaviors (e.g., helplessness and anhedonia; Grippo et al., 2009), but not anxiety (Grippo et al., 2012).

A recent body of research suggests that intranasal oxytocin spray (IN-OT) provides a non-invasive exogenous means to alter central levels of extracellular oxytocin in rodents (Neumann et al., 2013). The development of this spray as a pharmacologic treatment for an array of social disorders has generated great excitement because OT effectively modulates social behavior (DeMayo et al., 2017; but see Leng and Ludwig, 2016). Behavioral studies in rodents suggest that IN-OT treatment can alter social behavior. In prairie voles, for example, chronic IN-OT treatment increases social contact with sibling cage-mates (Bales et al., 2013), whereas medium and low doses (but not large doses) of IN-OT impair partner preference behavior in males (Bales et al., 2013), and such changes in behavior may be mediated by the effects of IN-OT on OTR density (Guoynes et al., 2018). Although, the acute use of IN-OT appears to be quite safe (DeMayo et al., 2017), few studies have investigated the potential long-term developmental impacts of IN-OT on social behavior (Bales and Perkeybile, 2012; but see Bales et al., 2013).

The circular nature of the potential for social environment to impact the OT system and for the OT system to impact social behavior (and thus the social environment) raises fundamental questions about the developmental mechanisms that shape adaptive behavioral repertories in adulthood. Moreover, because the social environment during development is complex and varies over time, interactions at different stages of development might create combinatorial interactions on behavioral and brain outcomes. We explored the dynamic interaction between early post-natal social environment, subadult social environment, and chronic dosing of extracellular OT *via* intranasal application on the social behavior of prairie voles at various stages of development. Several characteristics of prairie voles make the species particularly useful for the study of interactions between early social environments, social behavior, and the administration of OT. First, prairie voles in nature experience a wide variety of social experiences early in life, including single-mother and bi-parental rearing, and communal rearing in which older siblings alloparentally contribute to the care of younger siblings (Getz et al., 1981). As discussed above, simulating these social environments in the laboratory by removing fathers, and/or housing post-weaned animals in isolation or in groups alters the behavior in offspring (Grippo et al., 2007b; Ahern and Young, 2009; Pan et al., 2009; Prounis et al., 2015). Second, juvenile prairie voles in the lab readily engage in spontaneous alloparental care (care behavior towards novel unrelated pups; Roberts et al., 1998), and adult prairie voles form social attachments to opposite sex conspecifics (i.e., “partner preference behavior”; Getz et al., 1981; Williams et al., 1992). Last, these social behaviors are causally linked to OT function in the brain (Liu and Wang, 2003; Olazábal and Young, 2006a; Walum and Young, 2018).

In the current study, we hypothesized that the interaction between perinatal social environments (single-mother reared or bi parentally reared) and subadult social environments (isolated housing or group housing) would result in distinct expression of prosocial behavior during adolescence and adulthood. Furthermore, we predicted that IN-OT would increase the prosocial behavior of “socially limited” prairie voles (i.e., reared by single-mothers, followed by social isolation) to a greater extent than voles experiencing standard (and relatively enriched) rearing. This prediction was based on a previous study that showed “socially limited” voles exhibited higher densities of OTR in regions of the brain implicated in prosocial behavior, including the LS, prefrontal cortex (PFC), and basolateral amygdala (BLA; Prounis et al., 2015).

## Materials and Methods

### Animals and Early Life Manipulations

All subjects came from the first litter of breeding pairs created specifically for this experiment. The breeders originated from our colony of prairie voles, which were originally trapped in Champaign County, IL, USA. Animals were housed in standard polycarbonate rodent cages (29 × 18 × 13 cm) lined with Sani-chip bedding and provided nesting material, and kept on a 14:10 light-dark cycle. Animals were provided rodent chow (Laboratory Rodent Diet 5001, LabDiet, St. Louis, MO, USA) and water *ad libitum*. Ambient temperature was maintained at 20 ± 2°C. All procedures were approved by the Institutional Care and Use Committee of Cornell University (protocol number 2013-0102).

**Figure 1:**
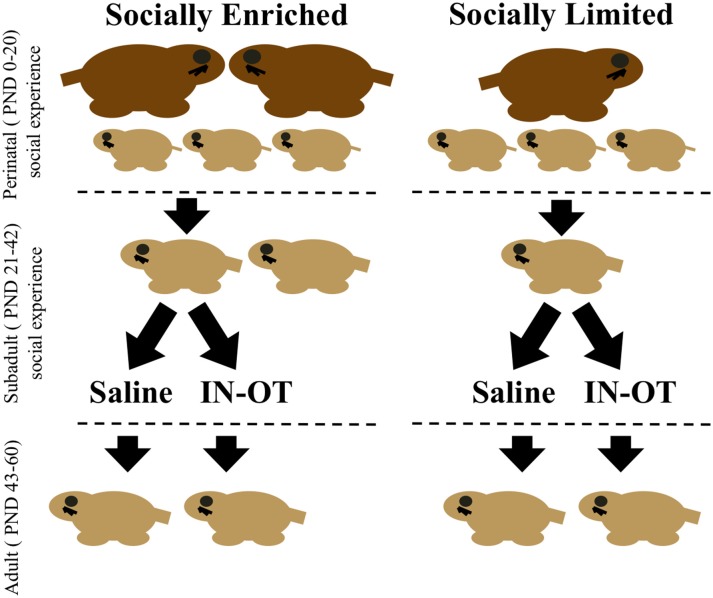
Timeline of social manipulations and intranasal treatment. Perinatal social manipulations occurred between postnatal day (PND) 0–20 and consisted of presence or absence of the father. Subadult social manipulations occurred between PND 21 (weaning) through PND 42 and consisted of being group-housed with a same-sex sibling or housed in isolation. During this period, intranasal saline, or intranasal oxytocin (IN-OT) was administered daily. Juvenile affiliation tests were conducted during this period. Adulthood was defined as beginning at PND 43 and lasted through the end of behavioral testing (PND 60), during which time alloparental care tests and partner preference tests were conducted.

All breeding units created for this experiment had litters culled to 3–5 pups. Male subjects were assigned to groups at birth that exposed them to one of two different social experiences across pre-weaning and post-weaning development. One group of “socially enriched” males was first reared by both a mother and father (i.e., a biparental family unit), and then group-housed with a male sibling at weaning (PND 21; Figure 1). The second group of “socially limited” males was reared by a single-mother after the father was removed on PND 0 and then housed in isolation at weaning (Figure 1). We use the terms “socially enriched” and “socially limited” to label the treatments in an overly simplistic way to convey that the social experiences we created incorporated social opportunities with more or fewer individuals, relative to each other. We do not intend for these terms to convey preconceived notions of one condition being “better” or more adaptive than the other. We have previously shown that these manipulations during both pre-weaning and post-weaning produce group differences in OTR expression and social cognition in male prairie voles (Prounis et al., 2015).

### Intranasal Oxytocin Treatments

Between PND 21 and 42, subjects received daily intranasal treatments of either saline or oxytocin (0.8 IU/kg) between 08:00 h and 12:00 h (Figure 1). This dose of chronic IN-OT treatment impacts social behavior of male prairie voles and is closely equivalent to a weight-adjusted dose commonly used in human studies (Bales et al., 2013). We applied a total of 25 μl of saline or OT from a pipette tip around the nasal cavity while the subject was scruffed and held belly-up, alternating sides so that 12.5 μl was applied to each nostril and resulting in inhalation of the solution. In total, four groups were created corresponding with both early-life manipulation and intranasal treatment: “socially enriched” + Saline, “socially enriched” + OT, “socially limited” + Saline, “socially limited” + OT (Figure 1). Subjects were weighed every week to determine the effects of the different conditions on body size and growth and to factor any mass differences into performance on behavioral tests.

### Behavioral Testing

Subjects performed a series of behavioral tests during and after the period of intranasal treatment. These included four juvenile affiliation tests, two alloparental care tests, and a partner preference test (see below).

#### Juvenile Affiliation Test

Immediately after intranasal treatment on PND 22, 28, 35, and 42, subjects were placed in a standard-sized cage (29 × 18 × 13 cm) for 30 min of acclimation prior to testing. After acclimation, unrelated and novel juvenile voles (between PND 15 and 21) were placed on the opposite end of the cage from the subject. We assessed the social contact time (defined as all non-agonistic physical contact) between subjects and the juvenile voles during the 10-min test. The timing of these tests correspond with expected release of oxytocin in the brain after intranasal treatment (Neumann et al., 2013). Thus, the weekly juvenile affiliation tests were intended to examine the immediate effects of IN-OT on prosocial behavior with a non-threatening stimulus animal, while also testing for changes in social responses over the weeks of chronic administration. The final sample sizes analyzed for all four juvenile affiliation tests were: “socially enriched” + Saline, *N* = 11; “socially enriched” + OT, *N* = 10; “socially limited” + Saline, *N* = 12; “socially limited” + OT, *N* = 12.

#### Alloparental Care Tests

On the day after the last intranasal treatment (PND 43), subjects performed the first of two alloparental care tests. Subjects were placed in a novel standard-sized cage (29 × 18 × 13 cm) to acclimate for 30 min. After acclimation, two unrelated neonates (between PND 2 and 5) were placed at the opposite end of the cage from the subject. The amount of huddling behavior and aggression was quantified during the 10-min test. Huddling behavior was scored as the total time the subject was stationary and completely covering at least one of the two stimulus pups in the test. Aggression was scored as any lunges and biting behavior. An experimenter watched the social interaction from a close distance but out of sight of the voles to ensure that if the subject behaved aggressively to the stimulus pup, the trial could be terminated before the stimulus animal was harmed. A second alloparental care test was implemented on PND 58, as just described. This allowed us to determine behavioral effects both immediately after and weeks after the period of chronic intranasal treatment. This schedule also allowed us to examine the effects of treatment on behavior at an age associated with subadult peripubertal animals (~PND 21–45) and adulthood (~PND > 45). There were two instances where the subject did not move from the corner of the cage at the start of the video (one during the PND 43 test, and one during the PND 58 test); these recordings were excluded from analysis. The final sample sizes analyzed for the PND 43 alloparental care test were: “socially enriched” + Saline, *N* = 11; “socially enriched” + OT, *N* = 10; “socially limited” + Saline, *N* = 12; “socially limited” + OT, *N* = 11. The final sample sizes analyzed for the PND 58 alloparental care test were: “socially enriched” + Saline, *N* = 11; “socially enriched” + OT, *N* = 9; “socially limited” + Saline, *N* = 12; “socially limited” + OT, *N* = 12.

#### Partner Preference Test

Last, on PND 60 subjects performed a partner preference test after 24 h of cohabitation with a sexually receptive female primed with dirty male bedding (Richmond and Stehn, 1976; Carter et al., 1980; Dluzen et al., 1981). To prime the females, urine-soaked bedding (taken from the cage of males that were unrelated to both the male subject and the female) was placed in the female’s home cage a day before animal pairing. The 24 h cohabitation time is sufficient to form partner preference in male prairie voles (DeVries and Carter, 1999; Blocker and Ophir, 2016). On the day of testing, we evaluated animals in the “partner preference test” following Williams et al. (1992). Briefly, the female partner and the unfamiliar female were tethered to opposite compartments of a three-compartment apparatus. After 30 min of acclimation, the subject male was placed in the unoccupied middle compartment from which it could freely move between all three compartments of the apparatus. An observer blind to treatment scored the amount of time the subject spent in side-by-side contact with both the female partner and with an unfamiliar sexually primed adult female over a 180-min test period. These times were compared to determine the degree of preference to cohabitate with the partner. Two recordings were unable to be analyzed due to technical issues. The final sample sizes analyzed for the partner preference test were: “socially enriched” + Saline, *N* = 11; “socially enriched” + OT, *N* = 9; “socially limited” + Saline, *N* = 11; “socially limited” + OT, *N* = 12.

### Data Analysis

All behavioral data were collected using Noldus Observer XT 14.0 (Noldus Information Technology Inc., Leesburg, VA, USA). For the juvenile affiliation tests, we performed a two-factor ANOVA to compare social contact time between groups according to social manipulation and intranasal treatment, and we used Tukey HSD tests to determine significant *post hoc* comparisons between groups. The findings from our initial two-factor ANOVA motivated us to perform a mixed factorial repeated measures ANOVA to compare changes in social contact time over the four timepoints between socially enriched and socially limited males. Due to non-normal distribution of data for proportion of huddling time in the alloparental care tests, we performed a Kruskal–Wallis test to detect significant group differences, and we used Dunn’s test for *post hoc* comparisons between groups. We performed a Pearson’s chi-square test for independence to compare the incidence of attack behavior in the alloparental care tests. We included subjects that attacked pups in the analysis of huddling time because we believe the attack behavior fairly represents an absence of care behavior and is critical to understanding group differences. Excluding subjects that attacked the pups from analysis of the alloparental care test would also severely reduce our sample size and result in a drastic loss of statistical power. Paired *t*-tests compared the side-by-side contact time with a partner female vs. an unfamiliar female for individual groups in the partner preference test. A two-factor ANOVA compared the preference score (contact with partner—contact with unfamiliar female) according to social manipulation and intranasal treatment, and Tukey HSD determined significant *post hoc* comparisons between groups. We performed a Pearson’s chi-square test for independence to compare the proportion of subjects displaying a partner preference in the partner preference test. Both chi-square tests (for the alloparental care test and the partner preference test) were limited to analysis of main effects (comparison based on social housing, or on intranasal treatment) due to sample size constraints when comparing all four groups. We considered an alpha ≤0.05 to be statistically significant for all tests. We report all *F* and *p*-values rounded to the nearest one-hundredth decimal place, except where *p* < 0.01, in which case we report them as such.

## Results

### Physical Development

No weight differences were found among males at PND 21 (ANOVA: *F*_(1,41)_ = 1.00, *p* = 0.32), but “socially limited” males weighed less than “socially enriched” males at PND 28 (*F*_(1,41)_ = 4.21, *p* = 0.05), PND 35 (*F*_(1,41)_ = 4.82, *p* = 0.03), and PND 42 (*F*_(1,41)_ = 4.73, *p* = 0.04). Intranasal OT treatment did not affect body mass at any stage of development. *Post hoc* comparisons showed no significant difference in body mass between any combination of the four groups (Tukey HSD: all *p’s* > 0.18). Body mass did not correlate with any behavioral test at the comparable age (see Supplementary Table S1).

### Juvenile Affiliation Tests

**Figure 2:**
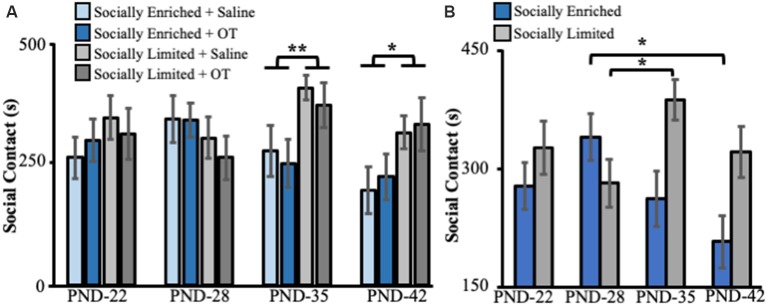
**(A)** Mean (±SE) seconds (s) subjects spent in social contact with novel juvenile voles (aged PND 15–21) as a function of early-life social experience and intranasal treatment on juvenile affiliation behavior in subadults. **(B)** Mean (±SE) seconds (s) subjects spent in social contact with novel juvenile voles (aged PND 15–21) as a function of only early-life social experience on juvenile affiliation behavior in subadults. OT, intranasal oxytocin treated animals; Saline, intranasal saline-treated animals; PND, postnatal day. *Indicates *p* < 0.05; **indicates *p* < 0.01.

Social rearing environment did not impact the amount of social contact time with juveniles at PND 22 (ANOVA: *F*_(1,41)_ = 1.10, *p* = 0.30) and PND 28 (*F*_(1,41)_ = 1.77, *p* = 0.19; Figure 2A). However, we found a main effect of social environment for tests performed on PND 35 (*F*_(1,41)_ = 8.12, *p* < 0.01) and PND 42 (*F*_(1,41)_ = 5.86, *p* = 0.02), with “socially limited” males having more social contact time than “socially enriched” males (Figure 2A). There was no effect of intranasal treatment on social contact with juveniles at any age (PND 22: *F*_(1,41)_ < 0.01, *p* = 0.98; PND 28: *F*_(1,41)_ = 0.27, *p* = 0.60; PND 35: *F*_(1,41)_ = 0.51, *p* = 0.48; PND 42: *F*_(1,41)_ = 0.22, *p* = 0.64). Likewise, there were no significant interactions between social environment and intranasal treatment at any age (PND 22: *F*_(1,41)_ = 0.53, *p* = 0.47; PND 28: *F*_(1,41)_ = 0.20, *p* = 0.66; PND 35: *F*_(1,41)_ = 0.02, *p* = 0.90; PND 42: *F*_(1,41)_ = 0.01, *p* = 0.92). *Post hoc* comparisons showed no differences between any of the four groups (Tukey HSD: all comparisons *p* > 0.08).

A mixed-effects ANOVA with a repeated measure for the age of testing revealed a significant interaction between developmental time and social rearing environment on social contact with juveniles (*F*_(3,129)_ = 4.43, *p* < 0.01, Figure 2B). Pairwise comparisons (with Bonferroni correction for multiple comparisons) showed that “socially enriched” males significantly reduced their social contact time with juveniles between PND 28 and PND 45 (*p* = 0.01, Figure 2B), whereas “socially limited” males increased their contact time with juveniles between PND 28 and PND 35 (*p* = 0.04, Figure 2B) and displayed comparable levels of juvenile contact between PND 28 and 45 (*p* = 0.99, Figure 2B).

### Alloparental Care Tests

**Figure 3:**
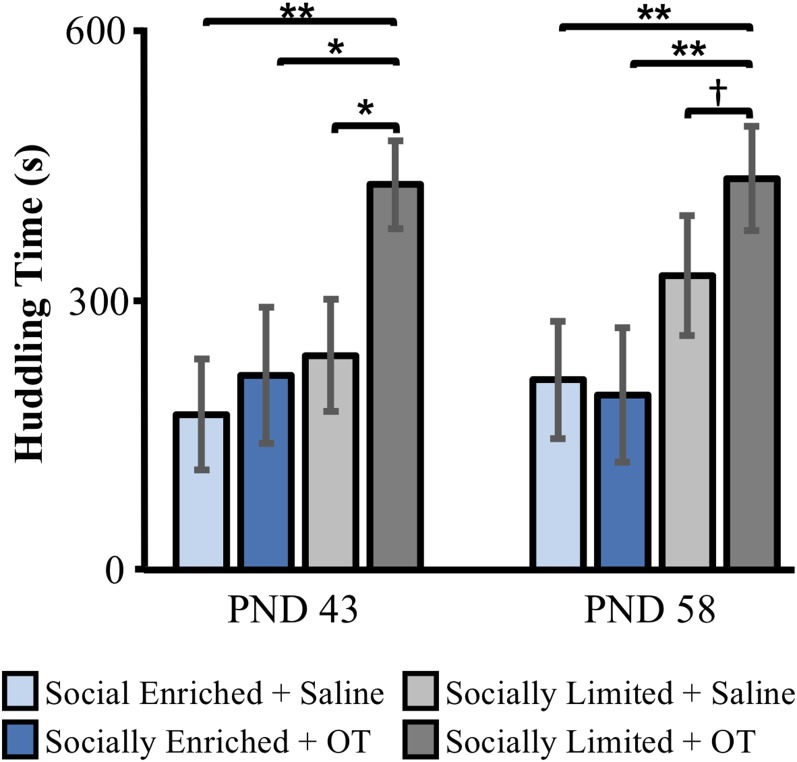
The effects of early-life social experience and intranasal treatment on huddling behavior in alloparental care tests at PND 43 and PND 58. Mean (±SE) time subjects spent huddling over at least one of two unrelated neonates (aged PND 2–5) is presented. OT, intranasal oxytocin treated animals; Saline, intranasal saline-treated animals. ^†^Indicates *p* = 0.06; *indicates *p* < 0.05; **indicates *p* < 0.01.

We found group differences in the proportion of time subjects huddled with pups at PND 43 in the first alloparental care test (Kruskal–Wallis: $x_{\text{(3)}}^{2}$ = 8.91, *p* = 0.03). *Post hoc* comparisons showed that “socially limited” + OT males spent more time huddling than “socially limited” + Saline males (Dunn’s test: *p* = 0.01), “socially enriched” + Saline males (*p* < 0.01), and “socially enriched” + OT males (*p* = 0.02; Figure 3). We found nearly the same group difference in the PND 58 alloparental care test ($x_{\text{(3)}}^{2}$ = 10.60, *p* = 0.01), with *post hoc* comparisons showing that “socially limited” + OT males spent more time huddling with pups than “socially enriched” + Saline (*p* < 0.01), and “socially enriched” + OT males (*p* < 0.01; Figure 3). A non-significant trend suggested “socially limited” + OT males might also spend more time huddling pups than “socially limited” + Saline males at PND 58 (*p* = 0.06; Figure 3).

At PND 43, immediate attack of pups during the test resulted in early termination of the test with zero proportion of time spent huddling scored for a subset of “socially enriched” + Saline males (*N* = 5, 45.5%), “socially enriched” + OT males (*N* = 2, 20%) and “socially limited” + Saline males (*N* = 3, 25%). Similar incidents of attack behavior were found at PND 58 (“socially enriched” + Saline: *N* = 4, 36.4%; “socially enriched” + OT: *N* = 1, 11.1%; “socially limited” + S: *N* = 2, 16.7%). At both PND 43 and PND 58, no “socially limited” + OT males attacked the pups. When comparing groups according to intranasal treatment, subjects receiving IN-OT (“socially limited” and “socially enriched” males combined) were less likely to attack than subjects receiving saline (“socially limited” and “socially enriched” males combined) at PND 43 ($x_{\text{(1)}}^{2}$ = 3.99, *p* = 0.05; Supplementary Table S2) and PND 58 ($x_{\text{(1)}}^{2}$ = 3.73, *p* = 0.05; Supplementary Table S3).

### Partner Preference Test

**Figure 4:**
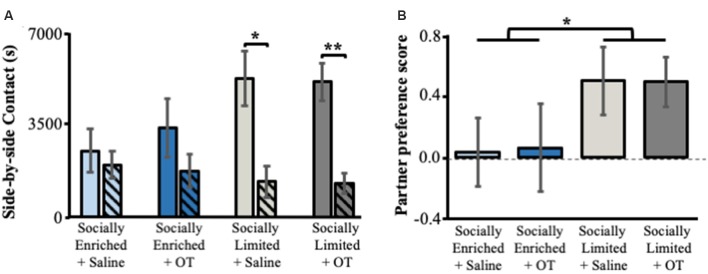
The effects of early-life social experience and intranasal treatment on partner preference behavior. **(A)** Mean (±SE) side-by-side contact in seconds (s) with the female partner (solid bars) and unfamiliar female (hatched bars). **(B)** Mean (±SE) partner preference score (time in seconds subjects spent in side-by-side contact with partner—time spent in side-by-side contact with the unfamiliar female/total time in side-by-side contact). Color scheme follows Figures 2, 3. OT, intranasal oxytocin treated animals; Saline, intranasal saline-treated animals. *Indicates *p* < 0.05; **indicates *p* < 0.01.

Only “socially limited” + OT (*t*_(11)_ = 3.89, *p* < 0.01) and “socially limited” + Saline (*t*_(10)_ = 2.53, *p* = 0.02) males demonstrated a significant preference for the female partner based on side-by-side contact time with each female in the partner preference test (Figure 4A). Surprisingly, a preference for side-by-side contact with the partner was not found for “socially enriched” + Saline (*t*_(10)_ = 0.48, *p* = 0.32) and “socially enriched” + OT males (*t*_(8)_ = 1.02, *p* = 0.17). Comparison of a partner preference score (side-by-side time with partner − side-by-side time with unfamiliar female/total side-by-side time) revealed a main effect of social environment, with “socially limited” males having larger preference scores than “socially enriched” males (ANOVA: *F*_(1,39)_ = 4.19, *p* = 0.05; Figure 4B). We performed an additional analysis of group differences in partner preference behavior by comparing the proportion of individuals demonstrating a preference for the partner, defined as the subject spending over 60% of total side-by-side contact with the partner. According to this approach, “socially limited” subjects (+OT and +Saline males combined) were more likely to form a preference than “socially enriched” subjects (+OT and +Saline males combined; chi-square test of independence: $x_{\text{(1)}}^{2}$ = 3.74, *p* = 0.05; Supplementary Table S4).

## Discussion

The combination of early social environment and IN-OT had variable effects depending on the developmental stage and the social behavior being tested. The social manipulation contrasted a relatively socially limited environment (no access to fathers before weaning or siblings after weaning) with a socially enriched environment (access to two parents and then a sibling). We found that males reared in “socially limited” environments engaged in more social contact with juveniles when they were subadults (PND 35 and PND 42) when compared to males reared in “socially enriched” environments. Furthermore, whereas “socially limited” males increased social contact with juveniles as they became older, males reared in “socially enriched” environments reduced social contact with juveniles as subadults. “Socially limited” males were also more likely to form a partner preference in adulthood when compared to “socially enriched” males. Lastly, chronic doses of IN-OT during post-weaning development led to a high degree of alloparental care behavior in “socially limited” males. This result suggests an additive or synergistic effect wherein a two-hit social deprivation treatment during postnatal development and IN-OT treatment promotes pro-social behavior in male prairie voles (see Ebitz and Platt, 2014).

Our findings indicate that, regardless of intranasal OT treatment, the combination of single-mother rearing during perinatal stages and isolated housing during subadult stages produced male prairie voles that: (i) engaged in more social contact in peri-adolescence (Figure 2); and (ii) developed stronger partner preferences in adulthood (Figure 4). Our results contribute to the small but growing number of reports that address the effects of perinatal and subadult social environments on prairie vole development. For example, prairie vole pups weigh more, open their eyes and grow hair sooner, and begin eating solid food and exploring outside the nest more rapidly when fathers were present (Wang and Novak, 1992, 1994), indicating that paternal care accelerates physical development of prairie vole offspring in the lab. Indeed, our data were consistent with this interpretation, showing that “socially enriched” males weighed more than “socially limited” males shortly after weaning and throughout subadulthood. Ahern and Young (2009) demonstrated that single-mother reared males did not form partner preferences after 1 day of cohabitation, instead, requiring a week of cohabitation before a preference was formed. This study also found no effect of rearing condition on alloparental care behavior in males, although females without fathers were less alloparental (Ahern and Young, 2009). Our results replicate this, finding no main effect of single-mother rearing on alloparental care in males (Figure 3). Surprisingly, our results in the partner preference test were inconsistent with Ahern and Young (2009), showing that single-mother reared males were more likely to form a partner preference than bi-parentally reared males. Similarly, unlike our study, Wang and Novak (1994) reported that time engaged in allogrooming and play was lower in males reared without fathers compared to those reared with both parents.

We suspect that these inconsistencies between our study and others could be attributable to elements of our experimental design. These differences could include the impact of social isolation at the subadult life stage (which was not investigated in these other studies), the unintended consequences of the necessary handling associated with the delivery of intranasal OT or Saline, or the combination of manipulating social experience at two important stages of development (see below).

First, several studies have found that pre-adult social isolation leads to an increase in social interaction (Wongwitdecha and Marsden, 1996; Pan et al., 2009; Gilles and Polston, 2017). For example, compared to group-housed males, male prairie voles that experience post-weaning social isolation prefer spending time in a cage containing a tethered novel male over an empty cage (Pan et al., 2009). Results such as these indicating that developmentally isolated animals become more social are consistent with our data in which “socially limited” animals increased social contact (alloparental care) with juveniles at PND 35 and PND 42.

Second, it is worth considering the ways in which the intranasal administration regimen might have impacted our animals. The handling that our animals experienced was extremely brief and they showed no observable evidence of discomfort or distress following each dosing. However, experimental scruffing of mothers increases maternal licking and grooming (Bales et al., 2007a) and could induce sub-threshold physiological reactions that cumulatively contribute to long-term effects on brain and behavior development. For example, we know that isolated male prairie voles have higher circulating concentrations of OT in their plasma, and higher activity of OT-reactive neurons in the paraventricular nucleus in response to experiencing a resident-intruder test (Grippo et al., 2007a), suggesting that social stress can induce endogenous release of OT in animals predisposed to be OT-reactive. It is worth noting that all of our animals experienced the same amount of handling for intranasal delivery during the subadult period (PND 21–45). However, if daily scruffing produced a subtler but similar reaction to resident-intruder tests and isolated males were more susceptible to this effect, then “socially limited” animals receiving IN-OT would have also experienced higher doses of OT than we had planned. Thus, the synergistic effects of experimental handling and early-life social experience, with the exogenous delivery of OT could have produced a heightened sensitivity to OT in “socially limited” + OT males in a way that none of the other groups in our study experienced. This might provide an explanation for the unique alloparental behavior we observed among “socially limited” + OT males. Interestingly, “socially limited” + OT males exhibited care towards pups in every individual trial. In contrast, approximately half of the control males (“socially enriched” + Saline) attacked the pups during the trial. Our data also show that IN-OT treatment reduced the likelihood of attack behavior during the alloparental care test (Supplementary Tables S2, S3). Thus, whether or not these effects are directly attributable to the handling the animals received, the interactions of complex early-life social experiences with OT exposure (endogenously released, exogenously released, or both) clearly produced an overarching prosocial (less-aggressive) phenotype in males.

Finally, it is also possible that group housing has protective or buffering effects on other developmental consequences resulting from being reared without fathers. Although the behavioral outcomes were quite different in how they interacted, we have reported results indicating that subadult housing conditions have the potential to alter the outcome of behavioral effects established by the absence of fathers in the perinatal nest (Prounis et al., 2015). Prounis et al. (2015) also found that “socially limited” reared males had greater expression of OTR in some forebrain areas (see below) suggesting that the sequence of single-mother rearing followed by social isolation produces a distinct social and neural phenotype. Indeed, social stress promotes prosocial behavior (e.g., adult social isolation, Perry et al., 2016), and this relationship is believed to be mediated by oxytocin (Taylor, 2006). We speculate that one outcome of reduced social opportunities during both perinatal and subadult stages might be increased motivation to seek social interaction when opportunities arise, which might buffer against the stress of social isolation. This unique combination of early social experiences might also facilitate prosocial behaviors in adulthood that resulted in the increased likelihood of pairbond formation in “socially limited” males. These possibilities highlight the importance of considering how social experiences at different life stages might interact to alter and, in some cases, rescue or even reverse behavioral phenotypes.

Because prairie voles reared in standard conditions typically exhibit partner preferences, the relative lack of partner preferences in “socially enriched” voles was unexpected (with the issues discussed above notwithstanding). Interestingly, Bales et al. (2013) demonstrated that standard reared male prairie voles (i.e., “socially enriched”) receiving IN-OT at the same dose that we used, also failed to form bonds. In this respect, our results complement and replicate this earlier result. And we note that our “socially enriched” + Saline and “socially enriched” + OT males also did not differ in juvenile affiliation or alloparental care behavior, just as was reported in Bales et al. (2013). However, this does not explain why our Saline treated “socially enriched” animals did not form bonds. “Typical” partner preference behavior in prairie voles has been shown to be highly variable (Vogel et al., 2018), and could be attributable to a number of factors including, for example, whether or not the pair produced fertilized embryos (Curtis, 2010). It is important to acknowledge that approximately half of our “socially enriched” subjects still exhibited a partner preference (Supplementary Table S4). Unfortunately, we did not directly examine the fertilization success of female-male pairs and it is possible that infertility contributed to the failure to find consistent pairbonding in this group.

Our study did not address the precise mechanism of action promoting alloparental care in “socially limited” + OT males, but previous research in prairie voles highlights some potential places to explore. OTR in the nucleus accumbens (NAc) is particularly high in juvenile prairie voles and is correlated with alloparental care (Olazábal and Young, 2006b), and mediates alloparental care (Olazábal and Young, 2006a). Notably, chronic IN-OT (at the same dose used in our study) has been reported to increase OTR in NAc in female, but not male, prairie voles (Guoynes et al., 2018). The effects of OT on OTR are notably dose-specific; the same study found no effect with lower or higher IN-OT doses (Guoynes et al., 2018). Furthermore, persistent application of OT agonists causes reduction of OTR *via* internalization (Gimpl and Fahrenholz, 2001). Therefore, it is possible that the “medium” chronic dose we used (and used by Bales et al., 2013; Guoynes et al., 2018) exerted a classic inverted U effect on behaviors that was high enough to alter behavior, but low enough to avoid inducing OTR internalization, in which case behavioral effects might have disappeared. Interestingly, the prosocial effect on social affiliation (i.e., huddling) was observed after IN-OT treatment had stopped, and this effect persisted for approximately 2 weeks (PND 43 and 58). This long-lasting effect of chronic IN-OT into adulthood may be due to the combination of limited access to social interaction during juvenile and subadult development, and the organizational effects resulting from administration of exogenous OT during subadult development. Ebitz and Platt (2014) provide a compelling argument for why the effects of exogenous OT might reduce the typical prioritization of social information at the expense of other information or goals. This can occasionally lead to counter intuitive but adaptive ways that prosociality might, or might not, be expressed, and can be attributable to how OT regulates the way the neural circuitry responsible for social behavior accesses information about the social environment. The administration of exogenous OT and its interactions with early-life social experiences certainly could have caused long-lasting organizational effects on the endogenous OT system, observable later in adulthood after IN-OT administration had stopped but modified and enhanced prosocial behavior persisted. Such effects could express themselves in the endogenous release patterns or functioning of OT or other aspects of the neurochemistry in the brain, leading to an altered prioritization of alloparental care, pairbonding or both. Indeed, earlier studies demonstrate the complex relationship between exogenous OT and behavior, showing highly variable outcomes of alloparental care behavior and partner preference behavior according to four different doses of OT given i.p. shortly after birth in female prairie voles (Bales et al., 2007b).

We previously showed that animals experiencing the same rearing conditions as those in our “socially limited” group exhibited significant increases of OTR in the LS, PFC, septohippocampal nucleus (SHi), and BLA when compared to control subjects (“socially enriched”; Prounis et al., 2015). With the exception of the SHi, all of these structures are important nodes of the Social-Decision-Making Network (SDMN; see O’Connell and Hofmann, 2011)—a network of structures that integrates processing of social information with reward circuitry to facilitate an animal’s ability to respond to and prioritize social information in their natural world. Indeed, OTR and vasopressin receptor expression, particularly within the SDMN, are highly plastic and sensitive to environmental and developmental forces (Prounis et al., 2018). Chronic IN-OT may have further developmentally impacted mechanisms that alter the social decision-making process. In this light, our multifaceted manipulations of early-life social experience and IN-OT exposure might have altered the way in which the SDMN, and potentially OT’s function therein, processed social behavior. Developmental effects on OTR or other factors that assert functional influence over the SDMN could have altered function of the SDMN globally, in effect, shifting animals’ valanced prioritization of the social value of pups. This hypothesis could explain why “socially limited” males reduced attack responses towards pups and instead demonstrated greater huddling with them. This could have been even further exaggerated as a result of chronic IN-OT. For example, IN-OT doubles extracellular levels of OT in the amygdala and hippocampus of rats (Neumann et al., 2013). This potential combination of increased exogenous OT and probable developmentally induced OTR upregulation in the SDMN of “socially limited” + OT males provides a plausible mechanism that could facilitate behavioral differences in this group. We believe that a greater understanding of complex brain phenotypes and the behaviors they generate can be found in a deeper appreciation of the SDMN—a network of structures that collectively regulate emergent properties of social behavior.

Social behavior is dynamically shaped by interactions between the early social environment and developing nonapeptide systems. We altered both the social environment and the OT system in developing prairie voles, and in doing so we identified that responses to intranasally administered oxytocin can be mediated by the degree of social opportunities experienced in perinatal and subadult development. Our results have translational application to exploring clinical use of IN-OT in children, adolescent, and adult humans. The effects of IN-OT on human behavior are remarkably inconsistent (Bartz et al., 2011; Keech et al., 2018). This generates controversy ranging from people who fear negative long-term effects of chronic IN-OT application, to people who doubt there are any effects at all (Young, 2013; Leng and Ludwig, 2016). We offer a potential source of variable responses to IN-OT, highlighting the shaping force of early-life social environments. Importantly, future studies must continue to explore how early life social environments across life stages interactively shape phenotypes. As this foundation of knowledge grows we can begin to form predictions about how physiological and neural systems adaptively tune to these early environments. Future research should test predictions of adaptive tuning by combining early-life manipulations in the lab with fitness outcomes in ecologically relevant and complex environments. Such efforts are likely to reveal that, in the proper context, phenotypic outcomes of socially limited contexts sometimes described as deprivation or even pathological can have adaptive or positive outcomes.

## Data Availability

The datasets generated for this study are available on request to the corresponding author.

## Ethics Statement

The animal study was reviewed and approved by Institutional Care and Use Committee of Cornell University (protocol number 2013-0102).

## Author Contributions

GP conceptualized the experiment, developed the experimental design, analyzed data, and wrote the manuscript. AO conceptualized the experiment, and wrote the manuscript.

## Conflict of Interest Statement

The authors declare that the research was conducted in the absence of any commercial or financial relationships that could be construed as a potential conflict of interest.
