# Supplementary material for: The Impact of Early Postnatal and Juvenile Social Environments on the Effects of Chronic Intranasal Oxytocin in the Prairie Vole
Source: Front Behav Neurosci. 2019 Sep 13;13:206. doi: 10.3389/fnbeh.2019.00206 (PMC6753389; doi:10.3389/fnbeh.2019.00206)
Supplement: Supplementary file 1 [file Table_1.DOCX]

**Supplemental Table S1.** Correlations between body mass and prosocial behavior for all subjects.

| **Weight, PND** |  | **Measurement, Test, PND age** |  | ***r* (df)** | ***p*** |
| --- | --- | --- | --- | --- | --- |
| Body Mass (g)  (PND 21) | x | Social Contact (sec)  in Juvenile Affiliation Test  (PND 22) |  | -0.04 (43) | 0.80 |
| Body Mass (g)  (PND 28) | x | Social Contact (sec)  in Juvenile Affiliation Test  (PND 28) |  | -0.11 (43) | 0.47 |
| Body Mass (g) (PND 35) | x | Social Contact (sec)  in Juvenile Affiliation Test  (PND 35) |  | 0.05 (43) | 0.77 |
| Body Mass (g) (PND 42) | x | Social Contact (sec)  in Juvenile Affiliation Test  (PND 42) |  | 0.29 (43) | 0.06 |
| Body Mass (g) (PND 42) | x | Huddling Time (sec)  in Spontaneous Alloparental Care Test  (PND 43) |  | -0.17 (42) | 0.27 |
| Body Mass (g) (PND 42) | x | Huddling Time (sec)  in Spontaneous Alloparental Care Test  (PND 58) |  | -0.14 (42) | 0.37 |
| Body Mass (g) (PND 42) | x | Proportion of time (sec) in side-by-side contact with partner  in Partner Preference Test  (PND 60) |  | -0.29 (41) | 0.06 |

**Supplemental Table S2.** Association between intranasal treatment and attack behavior in the Spontaneous Alloparental Care Test at postnatal day 43.

| **Intranasal Treatment** | **Attack (%)** | **No Attack (%)** | **n** | **χ^2^- statistic^a^ (df)** | ***p*** |
| --- | --- | --- | --- | --- | --- |
| OT | 2 (9.5) | 19 (90.5) | 21 | 3.99 (1) | 0.05 |
| Saline | 8 (35.8) | 15 (65.2) | 23 |  |  |
| ^a^Chi-square test for independence | | | | | |

**Supplemental Table S3.** Association between intranasal treatment and attack behavior in the spontaneous alloparental care test at postnatal day 58.

| **Intranasal Treatment** | **Attack (%)** | **No Attack (%)** | **n** | **χ^2^- statistic^a^ (df)** | ***p*** |
| --- | --- | --- | --- | --- | --- |
| OT | 1 (4.8) | 20 (95.2) | 21 | 3.73 (1) | 0.05 |
| Saline | 6 (26.1) | 17 (73.9) | 23 |  |  |
| ^a^Chi-square test for independence | | | | | |

**Supplemental Table S4.** Association between social manipulation and partner preference in the partner preference test.

| **Intranasal Treatment** | **Attack (%)** | **No Attack (%)** | **n** | **χ^2^- statistic^a^ (df)** | ***p*** |
| --- | --- | --- | --- | --- | --- |
| OT | 9 (45.0) | 11 (55.0) | 20 | 3.74 (1) | 0.05 |
| Saline | 17 (73.9) | 6 (26.1) | 23 |  |  |
| ^a^Chi-square test for independence | | | | | |
